# Intermittent exposure of cultured endothelial cells to physiologically relevant fructose concentrations has a profound impact on nitric oxide production and bioenergetics

**DOI:** 10.1371/journal.pone.0267675

**Published:** 2022-05-13

**Authors:** Maria Luisa Fiorello, Andrew T. Treweeke, David P. Macfarlane, Ian L. Megson

**Affiliations:** 1 Division of Biomedical Sciences, University of the Highlands & Islands, Inverness, United Kingdom; 2 Department of Diabetes, NHS Highland, Raigmore Hospital, Inverness, United Kingdom; National Center for Toxicological Research, UNITED STATES

## Abstract

Hyperglycaemia is known to induce endothelial dysfunction and changes in metabolic function, which could be implicated in diabetes-induced cardiovascular disease. To date, however, little is known about the impact of physiologically relevant concentrations of fructose on endothelial cells. A novel *in vitro* model was devised to establish the impact of substitution of a small proportion of glucose with an equal concentration (0.1 mM or 1 mM) of fructose on EA.hy926 endothelial cells during periodic carbohydrate “meals” superimposed on a normoglycaemic (5.5 mM) background. Parallel experiments were conducted using meals consisting of normoglycaemic glucose, intermediate glucose (12.5 mM) or profound hyperglycaemia (25 mM), each delivered for 2 h, with and without substituted fructose over 50 h. Outcome measures included nitrite as a surrogate marker of the mediator of healthy endothelial function, nitric oxide (NO), and a range of bioenergetic parameters using a metabolic analyser. Despite its relatively low proportion of carbohydrate load, intermittent fructose induced a substantial reduction (approximately 90%) in NO generation in cells treated with either concentration of fructose. Cell markers of oxidative stress were not altered by this treatment regimen. However, the cells experienced a marked increase in metabolic activity induced by fructose, irrespective of the glucose concentration delivered simultaneously in the “meals”. Indeed, glucose alone failed to induce any metabolic impact in this model. Key metabolic findings were a 2-fold increase in basal oxygen consumption rate and a similar change in extracellular acidification rate–a marker of glycolysis. Non-metabolic oxygen consumption also increased substantially in cells exposed to fructose. There was no difference between results with 0.1 mM fructose and those with 1 mM fructose. Low, physiologically relevant concentrations of fructose, delivered in a pattern that mimics mealtime consumption, had a profound impact on endothelial function and bioenergetics in an *in vitro* cell model. The results suggest that endothelial cells are exquisitely sensitive to circulating fructose; the potential ensuing dysfunction could have major implications for development of atherosclerotic disease associated with high fructose consumption.

## Introduction

A functional endothelium is essential for cardiovascular health [1]. The endothelium represents the interface between the blood and the surrounding tissues [2] and is therefore exposed to unbuffered variations in blood-borne nutrients. Hyperglycaemia, the hallmark of both type 1 and type 2 diabetes, induces endothelial dysfunction, leading to macro- and microvascular complications [3, 4]. Both persistent hyperglycaemia and intermittent glycaemic excursions are known to induce endothelial dysfunction [5–8], mediated by oxidative stress [9, 10], advanced glycated end-product (AGE) formation, epigenetic modifications [11–13] and generation of a pro-inflammatory phenotype [7, 14, 15]. The long-term impact of hyperglycaemia, even when followed by relative normoglycaemia, has been demonstrated in clinical trials, such as the Diabetes Control and Complications Trial/Epidemiology of Diabetes Interventions and Complications (DCCT/EDIC) and the UK Prospective Diabetes Study (UKPDS) [16] and in animal studies [17]. A recent *in vitro* study [18] showed how endothelial metabolism and redox status are rewired by long-term oversupply of glucose, leading to a more quiescent cellular state and decreased superoxide dismutase (SOD) activation, effects that were not reversible after 48 hours of glucose normalisation.

The monosaccharide, fructose, is commonly ingested with glucose in the Western diet, either as part of the disaccharide, sucrose, or in high fructose corn syrup, fruit and honey [19]. Although fructose was once considered to be a desirable substitute for glucose in diabetes patients due to its low glycaemic index (GI) (GI = 23, versus GI = 100 for glucose) [20–22], the increase in fructose consumption over the last 40 years has instead been associated with an increase in metabolic syndrome [23, 24]. Whilst this effect has been postulated to be in part related to an overall increase in energy intake, there is clear evidence that fructose can directly lead to adverse metabolic effects. The liver is responsible for the metabolism of approximately 70% of the total fructose absorbed [25] and, conversely to glucose, hepatic fructose metabolism is unregulated, leading to adverse effects, such as increased *de novo* lipogenesis [26, 27], AGE formation [28–30], cellular ATP depletion and an increase in uric acid generation [31–33]. These aspects are exacerbated in the presence of other risk factors for poor health, such as type 2 diabetes [34, 35], leading to a greater risk of cardiovascular complications. Contemporary administration of fructose and glucose leads to increased risk of developing non-alcoholic fatty liver disease and dyslipidaemia [26, 36, 37], by reducing glucose oxidation and gluconeogenesis, while increasing metabolic intermediates that fuel *de novo* lipogenesis (DNL) and the triglyceride precursor, glycerol-3-phosphate [38, 39], as well as gene expression and activation of lipogenic enzymes [40, 41].

Fructose can reach concentrations of up to 1 mM post-prandially [42, 43]. Recent studies have identified inflammation, inhibition of nitric oxide (NO) production [44], a prothrombotic phenotype [45], oxidative stress [46], increased endothelial cell senescence [47] and augmented formation of intracellular and extracellular AGEs [28] to be the most important effects associated with fructose consumption. However, there is currently a lack of understanding of the impact of systemic fructose on endothelial cells, and little is known about the capacity of endothelial cells.

This study set out to investigate the effect of combined exposure of cultured endothelial cells to glucose and fructose, delivered at physiologically relevant concentrations and frequencies. It was hypothesised that fructose exposure detrimentally affects the availability of NO and alters metabolism in endothelial cells.

Cell culture has a great deal to offer this subject area, but equally presents a number of challenges with respect to recreating scenarios that accurately reflect the *in vivo* situation. For example, cells are often traditionally grown under very high glycaemic conditions (25 mM) that would rarely be found in even the most poorly controlled patient with diabetes. Additionally, in the vast majority of *in vitro* experimental models, alterations in glucose conditions applied to cells are continued for many hours or days that do not reflect post-prandial excursions, while glucose-induced changes in osmolarity are not often mitigated. In an extension to our previous endothelial model for hyperglycaemia [18], this study implemented a novel model that was designed to more closely mimic postprandial glucose oscillations in cells grown through multiple passages under normo-glycaemic conditions, whilst at the same time maintaining constant osmolarity by balancing with mannitol.

## Materials and methods

### Materials

Unless stated otherwise, all chemicals were purchased from Sigma Aldrich (Dorset, UK). Cell culture consumables were purchased from VWR (Lutterworth, UK).

### Cell culture and treatments

The endothelial cell line, EA.hy926 (ATCC CRL-2922; a commercially available fusion of primary human umbilical vein cells with a clone of A549 cells), was cultured in Dulbecco’s Modified Eagle’s Medium (DMEM; HyClone) containing 5.5 mM glucose, 2 mM L-glutamine, 5% penicillin/streptomycin (10000 Units/mL and 10 mg/mL) and 10% foetal bovine serum, in a humidified atmosphere at 37°C and 5% CO_2_. Cells pre-conditioned to 5.5 mM glucose through several (>5) passages were chosen for this study because those grown under traditional conditions (25 mM) glucose were previously found to be refractory to acute glycaemic changes [18]. Cells were seeded at 2x10^4^ cells/well in standard 96 well plates for nitrite and ROS analysis and in specialised Seahorse XF96 cell culture 96-well microplates for metabolic analysis. In both cases, cells were incubated for 12 hours (37°C, 5% CO_2_) to set down prior to initiation of the experimental protocol.

### Glucose and [glucose+fructose] treatments

#### Glucose only model

**Fig 1:**
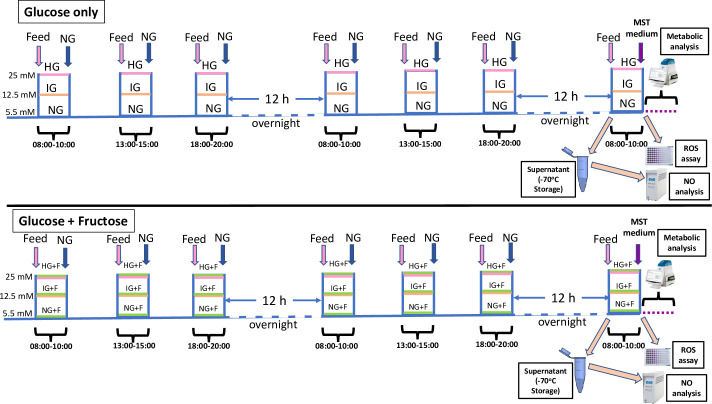
Representation of the experimental regimens for EA.hy926 cells (glucose only model–top panel; glucose+fructose–bottom panel). The total treatment time was 50 h, incorporating 7 treatment periods at intervals designed to mimic popular mealtimes in human populations. Two parallel sets of experiments were undertaken: cells destined for metabolic analysis were cultured in specialized 96 well microplates for this purpose; analysis was undertaken at the end of the 50 hour treatment period. Cells destined for ROS/superoxide analysis were cultured in standard 96 well microplates. Supernatant was harvested after 48 h for nitrite analysis, while ROS/superoxide analysis was conducted on the cells after the final 2 h meal exposure.

In the basic, glucose only model, cells preconditioned to normoglycaemic (NG; 5.5 mM) conditions were exposed to intermediate hyperglycaemia (IG; 12.5 mM) and extreme hyperglycaemia (HG; 25 mM) as the exclusive carbohydrate source in intermittent exposures of 2 hours each, delivered at times designed to mimic breakfast (08:00–10:00), lunch (13:00–15:00) and dinner (18:00–20:00), interspersed with episodes of normoglycaemia. This regime was implemented for up to 50 hours, with two overnight (20:00–08:00) “fasts”, during which NG conditions were maintained (Fig 1). A NG control regimen was included to represent exceptional glycaemic control and iso-osmolarity was maintained throughout with a balance of mannitol (Table 1). For practical reasons, supernatant for nitrite was taken after the second 12 h overnight fast to ensure accumulation of sufficient nitrite for measurement; the cells in these plates, however, went on to a further meal exposure and were then used for ROS/superoxide analysis. Parallel experiments conducted in specialised Seahorse XF96 cell culture 96-well microplates for metabolic analysis were also exposed to the final mealtime carbohydrate exposure before undergoing the mito- stress test outlined below.

**Table 1:** Composition of test glucose and glucose/fructose medium for the intermittent treatment.

Total carbohydrate concentration	Glucose only	Glucose concentration with 0.1 mM fructose	Glucose concentration with 1 mM fructose	Osmolarity control (mannitol)
**NG (5.5 mM)**	5.5 mM	5.4 mM	4.5 mM	19.5 mM
**IG (12.5 mM)**	12.5 mM	12.4 mM	11.5 mM	12.5 mM
**HG (25 mM)**	25 mM	24.9 mM	24 mM	0 mM

NG: normal glucose; IG: intermediate glucose; HG—hyperglycaemic

#### Glucose + fructose model

To test the impact of physiologically relevant substitution of a component of glucose load with fructose, a modification of the basic glucose only protocol was employed. Here, cells preconditioned under normoglycaemic conditions were exposed to the same pattern of hyperglycaemic episodes, but this time a portion (0.1 mM or 1 mM) of the glucose was replaced with fructose (Table 1). The concept was to mimic mealtimes that included fructose ingestion. All other aspects of the experiments were identical to the basic model, including interspersed periods of normoglycaemia (without fructose), an overnight fast, iso-osmolarity maintained through balanced mannitol and a normoglycaemic control arm, in which mealtime treatments consisted of 4.5 mM glucose + 1 mM fructose or 5.4 mM glucose + 0.1 mM fructose. In keeping with our previous work, cells typically reached confluence during the 48 h treatment regimens.

### Outcome measures

#### Nitrite accumulation

Supernatant from cells in both models was collected after the overnight fast (i.e. 12 h of accumulation) for nitrite analysis, by way of a marker of nitric oxide (NO) production using a Sievers 280i nitric oxide analyser (NOA). Medium from a triplicate for each condition (n = 9–14 independent experiments for each) was pooled into a single tube and stored at -80°C. Prior to analysis, batched test samples were defrosted on ice and diluted 1 in 4 in deionized water. For each sample, 100 μl was injected into the NOA chamber containing 4.5 ml of glacial acetic acid (Fisher Scientific), 100 μl of dilute antifoam B emulsion and 0.5 ml of sodium iodide (25 mg/ml; Fisher Scientific). Medium only samples for each condition were used as background controls. Data were acquired using Liquid Software (Version 2), integrated and the sample nitrite values extrapolated from the standard curve.

#### Metabolic analysis

The bioenergetic phenotype of the cells at the end of the treatment period was determined using a Seahorse Bioscience XF^e^96 Extracellular Flux Analyzer (Agilent) to measure both the oxygen consumption rate (OCR) and the extracellular acidification rate (ECAR) using the Seahorse mito-stress test (MST; Agilent). The assay was performed according to the manufacturer’s instructions and all Seahorse-related kits, reagents and consumables were purchased from Agilent unless stated.

The EA.hy926 cells were observed to be fully confluent at the end of the 50 hour treatment period and immediately prior to the MST assay. Medium was removed from each well, the cells washed twice with Seahorse assay medium (Seahorse XF Base Medium containing 1 mM pyruvate, 2 mM L-glutamine and 10 mM glucose, adjusted to pH 7.4), prior to incubation at 37°C for 45 minutes in a CO_2_-free incubator during which basal and then real-time changes in OCR and ECAR were monitored following three sequential injections of mitochondrial electron transport chain (ETC) modulators: first, oligomycin (1 μM; complex V inhibitor); next carbonyl cyanide-*4*-(trifluoromethoxy) phenylhydrazone (FCCP, 1 μM; an uncoupler of mitochondrial oxidative phosphorylation) and finally a combination of rotenone and antimycin A (both 0.5 μM), to inhibit complex I and complex III respectively.

All injectable treatments were freshly prepared, loaded into the injection ports and the sample plate loaded into the Seahorse XF^e^96 for analysis. Data aquisition started from the third measurement of each cycle and was analysed using Seahorse Wave Software (Version 2.6.0.31).

At the end of the assay, the protein content of each well was quantified using a Coomassie-Bradford-Protein Assay Kit (Merck, Sigma Aldrich), to allow for normalisation of data per mg of protein present in the well. Cells were lysed *in situ* with distilled water and repeated freeze-thaw cycles. This protein estimate assay was subsequently used as a surrogate for cell density where removal of cells for counting was not practical.

#### Total reactive oxygen species-reactive nitrogen species (ROS-RNS) assay

The total ROS-RNS and superoxide detection fluorescent assay (Enzo Life Sciences, UK) was used to estimate the ROS-RNS production of EA.hy926 cells seeded in a 96-well plate (2×10^4^ cells/well) and treated for 50 hours with NG, IG or HG medium, according to the protocol illustrated in Fig 1. Pyocyanin was used as a positive control for induction of ROS (200 μM for 20 min) and all treatments were made in triplicate. The assay was performed according to the kit instructions and the fluorescent signal detected (excitation/emission λ = 488/520 nm) using a VarioScan plate reader (Thermo-Fisher) with SkanIt Software 2.4.5 (Varioskan Flash). All results were normalized to the protein content of each well.

### Statistics

All data are expressed as mean ± SD unless otherwise stated; individual points on each graph indicate independent experiments. Data were analysed using appropriate parametric and non-parametric tests, as defined by normality (D’Agostino and Pearson test) and equal variance testing. Significant difference between means was accepted at P<0.05. Statistical tests were performed using GraphPad Prism version 6.00 software (GraphPad Software, San Diego, CA). Details of tests used are included in each figure legend; data were treated as unpaired in all data sets.

## Results

### Effect of glucose/fructose exposure on NO generation, index of endothelial cell function

**Fig 2:**
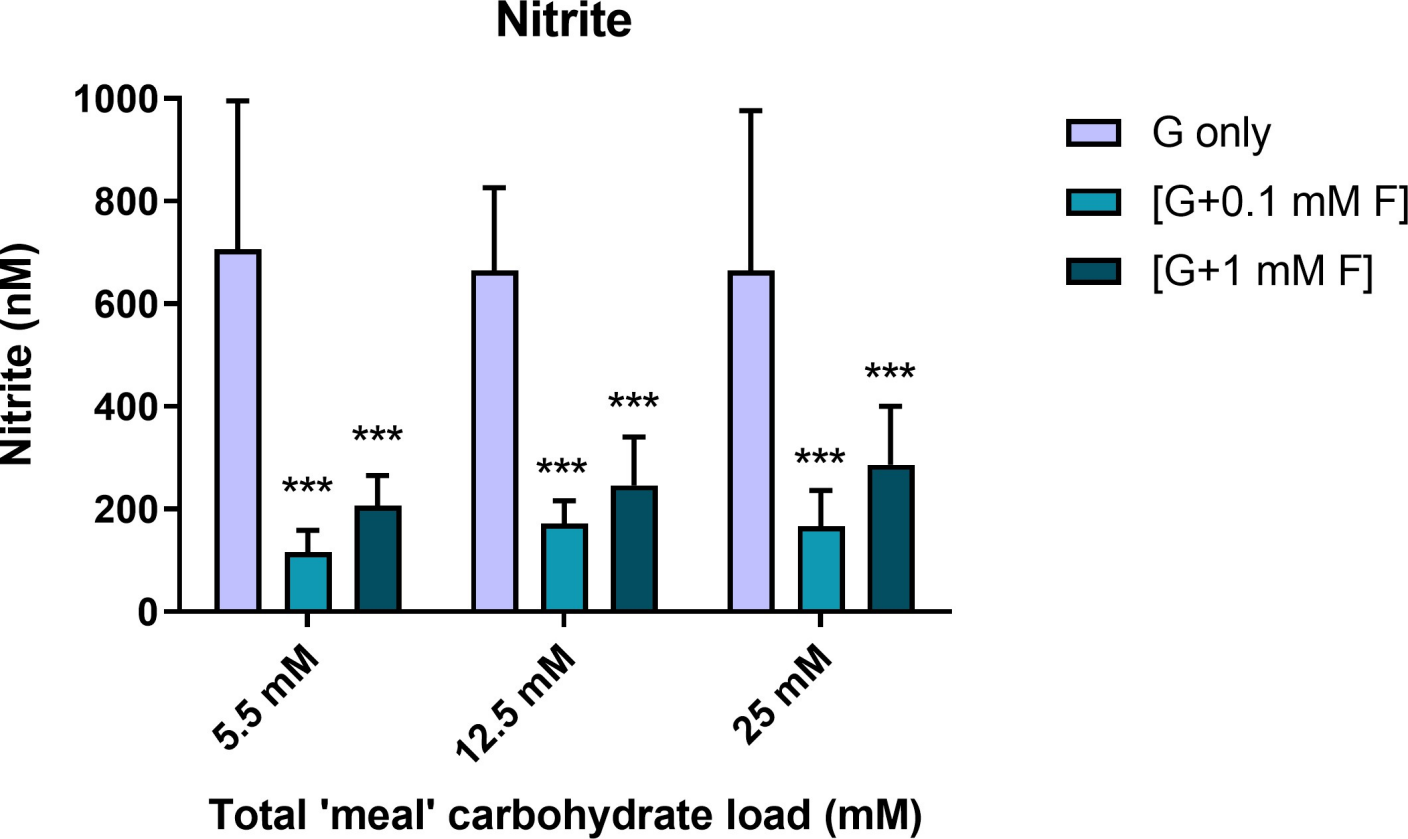
Nitrite accumulation as a surrogate for NO generation. Effect of intermittent glucose and glucose/fructose exposure on nitrite accumulation at NG, IG and HG test conditions (n = 12–14; ***P<0.001 Bonferroni post-test compared to glucose only control at equivalent concentrations).

Inclusion of fructose (both 0.1 mM and 1 mM) during intermittent exposure caused a profound decrease in nitrite accumulation, irrespective of the glucose concentration (Fig 2), despite relatively high variance in some of the datasets, which is not unusual for NO assessments. There was no significant difference between the data from experiments involving 0.1 mM fructose compared to 1 mM fructose (P>0.05; 2 Factor ANOVA with Bonferroni post-test). It is important to note that the nitrite measured was accumulated nitrite over the 12 h overnight period with 5.5 mM glucose and no fructose present. In addition, exposure to intermittent hyperglycaemia (12.5 mM or 25 mM) failed to have any impact on overnight nitrite accumulation.

### Effect of glucose/fructose exposure on cell bioenergetics

**Fig 3:**
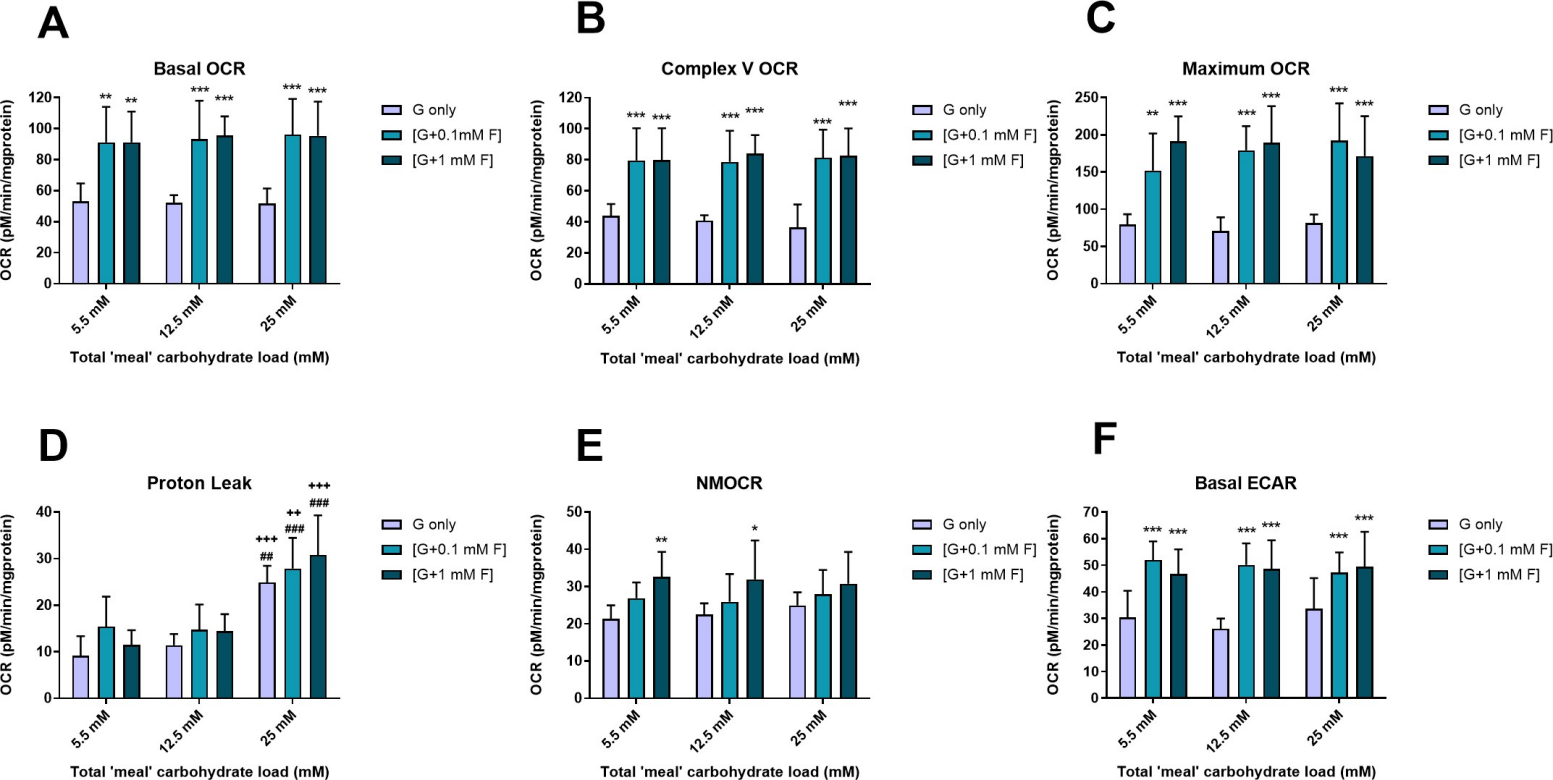
Cellular bioenergetics. Effect of intermittent glucose (G only)/glucose+fructose (G+0.1F, G+1F) exposure on cellular bioenergetics, described by (A) basal OCR, (B) complex V OCR, (C) maximal respiration, (D) proton leak, (E) NMOCR and (F) ECAR. Data are shown as mean ±SD for n = 6–8 separate experiments. Means are compared using 2-factor ANOVA with Bonferroni post-test. ***P<0.001; **P<0.01 for fructose-treated cells compared to glucose only treated cells with the same carbohydrate load. For proton leak, there was a significant impact of carbohydrate load (P<0.001) and of fructose (main effect, P<0.05). For D only: ^+++^P<0.001 and ^++^P<0.01 v equivalent bar at 5.5 mM load; ^###^P<0.001 and ^##^P<0.01 v equivalent bar at 12.5 mM load.

Overall, cells treated with the glucose/fructose combinations showed a marked increase in oxygen consumption over cells treated with glucose alone (Fig 3). At each of the three carbohydrate concentrations, basal OCR (Fig 3A), complex V OCR (Fig 3B) and maximum OCR (Fig 3C) values were markedly elevated to a similar extent (approximately 2-fold) for both 0.1 and 1 mM fructose substituation of glucose compared to glucose only treatments. Increasing glucose alone during the loading periods had no significant effect on any of these parameters (P>0.05; 2-factor ANOVA). By contrast, proton leak increased with glucose concentration during loading episodes (2.5-fold between 5.5 mM and 25 mM), whereas fructose had only a small but significant (P<0.05; two-factor ANOVA) effect (Fig 3D). NMOCR was increased only with 1 mM substituted fructose treatments compared to equivalent glucose only treatments (~1.5 fold increase; Fig 3E). Fructose substititution of glucose also caused an increase in basal ECAR (1.5–2-fold) for all combinations (Fig 3F); there was no differential impact of 0.1 mM and 1 mM fructose on ECAR. These data suggest that overall, the presence of even relatively low concentrations of fructose induces greater oxygen consumption and glycolysis, irrespective of the glucose concentration co-existing during the periodic treatments.

**Fig 4:**
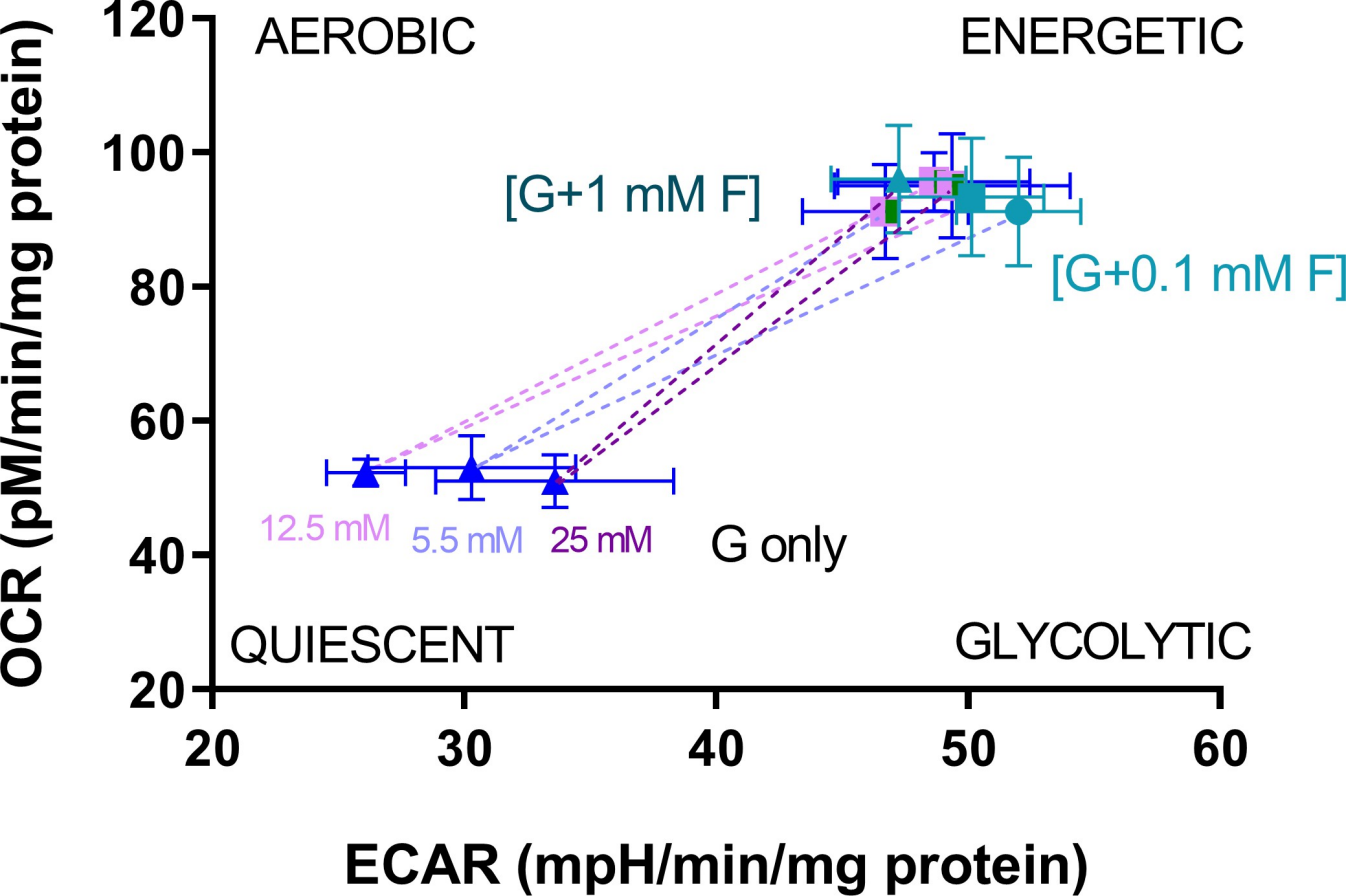
Energy map. Energy map for cells treated with intermittent glucose and glucose/fructose. Dotted lines represent the direction of change induced by fructose substitution for glucose (0.1 mM–light symbols; or 1 mM–dark symbols) in cells treated with different glucose concentrations. Data are expressed as mean ± SEM (n = 8–12 for each mean depicted).

The mean values of basal OCR and basal ECAR were used to create energy maps to identify changes in cell bioenergetic phenotype (Fig 4), which shows that intermittent glucose/fructose treatment generated a more ‘energetic’ metabolic phenotype (increased oxygen consumption and glycolysis) compared to those treated with intermittent glucose only; this was evident for NG, IG and HG treatments, using both fructose concentrations (Fig 3). Specifically, the presence of fructose resulted in a shift along the energetic/quiescent axis, but not the aerobic/glycolytic axis. There was no detectable impact of intermittent glucose excursions in the absenc of fructose.

### Effect of glucose/fructose exposure on measures of oxidative stress-associated parameters

**Fig 5:**
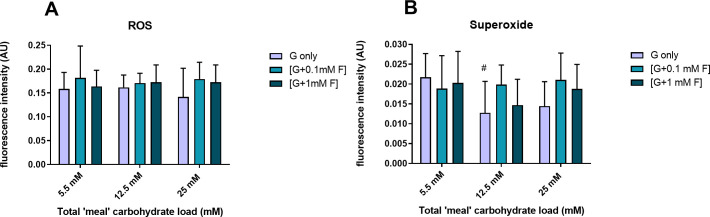
ROS generation. Effect of intermittent glucose and glucose/fructose exposure on total ROS-RNS (A) and superoxide (B; n = 7–12; #P<0.05 compared to 5.5 mM glucose alone).

The total ROS-RNS for cells subjected to intermittent glucose exposure had similar values under NG, IG and HG intermittent treatments (0.15 ± 0.03, 0.14 ± 0.03 and 0.17 ± 0.03 AU/mg protein respectively; Fig 5A; P>0.05). Similarly, superoxide was only modulated by intermittent glucose exposure to IG (Fig 5B). Inclusion of fructose (0.1 mM, 1 mM) with glucose (NG, IG, HG) had no significant impact on ROS/RNS or superoxide (Fig 5).

## Discussion

This study utilised a novel experimental intermittent carbohydrate exposure model, designed to mimic diurnal glycaemic oscillations. The data presented show that endothelial exposure to physiologically relevant concentrations of fructose had a profound impact on NO generation and cellular metabolism that was not replicated by intermittent hyperglycaemia in the absence of fructose in the same model. The results highlight the potential importance of post-prandial fructose overspill from first-pass hepatic clearance on endothelial function and bio-energetics, independent of any potential indirect effects of fructose (e.g. dyslipidaemia).

### Endothelial function

NO is a key determinant of endothelial function and was therefore measured in this study in the form of its surrogate, nitrite. The results showed that intermittent exposure to 0.1 and 1 mM fructose caused a profound inhibition (approximately 80–90% reduction) of nitrite accumulation, irrespective of the underlying glucose concentration. The lack of impact of the same treatment on ROS and superoxide generation suggests that the effect is not mediated by oxidative loss to peroxynitrite; instead it points to dysfunctional eNOS or pertinent activation pathways, including inhibitory post-translational modifications (such as excessive glycation) or reduced availability of NADPH, Ca^2+^ or tetrahydrobiopterin, necessary for eNOS-mediated NO synthesis [44, 48, 49]. What is clear is that the effect was not reversed by a 12 h interval between removal of fructose and collection of supernatant for nitrite analysis, suggesting that if the effect is replicated *in vivo*, then these data show a potentially deleterious consequence associated with fructose overspill into the vascular system, which could impair cardiovascular health by increasing atherogenic risk [50–52]. Of note is that intermittent hyperglycaemia induced by glucose alone failed to impact NO at all. This is contrary to our previous findings involving continuous exposure to 25 mM glucose over a 48 h period, whereby an inhibition of similar magnitude to that found for fructose was observed [18]. It seems, therefore, that both glucose and fructose have the capability to reduce NO synthesis substantially, but that fructose can effect this impact at considerably lower concentrations and reduced exposure time than is the case for glucose.

### Bioenergetics

The energy map clearly indicates that cellular exposure to fructose resulted in cellular metabolic activation, with an increase in both oxidative phosphorylation (increased OCR, most notably complex V-related OCR) and glycolysis (increased ECAR).

The increase in maximal OCR achieved with FCCP-mediated disruption of the proton gradient across the inner mitochondrial membrane reflects an increase in cellular mitochondrial efficiency, an adaptation mechanism activated in response to the enhanced electron flux from glucose/fructose treatment via glycolysis and the tricarboxylic acid (TCA) cycle. This increase in maximal OCR could be achieved by either greater transcription of genes encoding ETC complexes, post-translational activation of the complexes, or mitochondrial biogenesis [53]. This mechanism could maintain an optimal level of ROS, allowing more mitochondria to share the electron load in the form of reducing equivalents, (NADH and FADH_2_) during glycolysis and in the TCA cycle [54], a finding that has been identified previously in a similar cell culture models [18, 56] and attributed to the Crabtree effect [55–57].

It is not clear whether an increase in aerobic metabolic activity observed with intermittent fructose exposure would be deleterious *in vivo*. However, due to the glycolytic nature of endothelial cells, dysregulation of cellular metabolism is likely to represent a disturbance in the homeostatic state of the cells. For example, an increase in metabolism in endothelial cells has been observed in atherosclerosis, as well as during angiogenesis [58, 59]. The switch to more aerobic glucose and fructose metabolism could represent a functional disadvantage for the endothelium; greater oxygen consumption or requirement could deprive oxygen release to the perivascular tissues and compromise the ability of endothelial cells to adapt to a less oxygenated environment. Fructose-induced dysregulation of cell metabolism could also result in an imbalance of intracellular redox state, which could, in turn, affect the ability of endothelial cells to produce NO.

There are a number of mechanisms by which fructose and its metabolites may directly and indirectly influence intracellular bioenergetics and function, including provision of fructose-6-phosphate (F6P) substrate for the aerobic, hexosamine and pentose phosphate pathways. Given that the total carbohydrate load was not different between the glucose only and glucose/fructose arms of this study, and fructose represented only a small fraction of this load, it seems implausible that increased substrate provision is responsible for the substantial changes in metabolism witnessed.

Unregulated fructose phosphorylation leads to ATP depletion [32, 60]. A consequence of this ATP depletion is adenosine monophosphate-activated protein kinase (AMPK) activation [61, 62], which drives an increase in glycolysis and fatty acid oxidation to restore ATP concentrations [63, 64]. As such, the higher mitochondrial oxygen consumption in the presence of fructose (shown by the increase in basal OCR, complex V OCR and maximal OCR) could reflect AMPK activation, in an attempt to restore cellular energy balance. Moreover, ATP depletion and the consequent increase in AMP has been shown to link to uric acid formation, through activation of xanthine oxidase (XO) [65], which consumes oxygen, and depresses NO production at the same time as enhancing superoxide, hydrogen peroxide and hydroxyl radical generation [66, 67]. Accordingly, the XO inhibitor, allopurinol, has been shown to improve cardiovascular outcomes in patients with type 1 and type 2 diabetes [68, 69] and in hyperuricaemic patients [70, 71].

Fructose-6P can be further phosphorylated in position 2 by phosphofructokinase-2/fructose bisphosphatase-2 (PFK2/FBPase-2). The product, fructose 2,6-biphosphate allosterically activates phosphofructokinase 1 (PFK1) [72], one of the key glycolytic enzymes, resulting in greater production of glycolytic metabolic intermediates, like triose phosphates (glyceraldehyde-3-phosphate and dihydroxyacetone phosphate). Such products can be further metabolised, via TCA and oxidative phosphorylation, or can participate in alternative glucose metabolic pathways, such as the pentose phosphate pathway and the hexosamine pathway.

Glucose-6-phosphate dehydrogenase (G6PD), the rate limiting enzyme of the oxidate branch of the pentose phosphate pathway, is important for the production of NADPH [73], a cofactor for eNOS-mediated NO synthesis. Therefore an increase in glycolytic intermediates, such as F6P and glyceraldehyde-3-phosphate, and an associated inhibition of G6PD, has the potential to the decrease NO production via this mechanism. In addition, increased flux of F6P through the hexosamine pathway can produce UDP-N-acetylglucosamine (UDPGlcNAc), a substrate for protein regulation by O-linked glycosylation [74]. Excessive protein O-linked glycosylation can, in turn, contribute to eNOS inactivation [75].

### Oxidative species production

Proton leak was the only parameter measured in these experiments that increased with intermittent glucose, but was not significantly altered by the presence of fructose. Proton leak is a well-known phenomenon that can be induced by activation of uncoupling proteins, allowing protons to leak back across the inner mitochondrial membrane, reducing the protonmotive force essential for efficient ATP synthase (complex V) activity and uncoupling oxygen utilisation and ATP production. Proton leak is a contributor in endothelial activation in early atherosclerosis [76] is one scenario in which increased, although exposure of endothelial cells to oxidative stress is understood to reduce proton leak. The prevailing hypothesis linking proton leak to oxidative stress is that uncoupling reduces ROS generation, perhaps by way of a protective mechanism. Our results perhaps hint at reduced superoxide in cells exposed to hyperglycaemia (12.5 mM in particular) with or without fructose, but a convincing inverse relationship between glycaemic load and ROS is not clear in this model.

NMOCR is another potential indicator of oxidative stress on account of utilisation of oxygen by ROS-generating enzymes such as cyclo-oxygenases, lipoxygenases and NAD(P)H oxidases. Our results indicate that intermittent exposure to fructose at both 0.1 mM and 1 mM has the capability of greatly increasing NMOCR (~1.5-fold), but this does not correlate with the ROS/superoxide data, which were inconclusive with respect to the impact of fructose.

In interpreting these data, it is important to consider that the cells were austensibly healthy and likely capable of mounting an effective counter-regulatory defence in response to an acute, potentially low-level rise in ROS generation. The impact of more prolonged glycaemic insult, or that in cells already in a state of disease-induced oxidative stress, might be more substantial in terms of oxidative damage. The finding that alterations in proton leak (hyperglycaemia) and NMOCR (fructose) are induced in endothelial cells, at least illustrates that there is potential for dysregulation-induced redox impacts.

### Limitations

Use of an immortalized cell line (EA.hy926) could be seen as a limitation of this study, but was deemed essential because, unlike primary endothelial cells, they are resistant to senescence and de-differentiation through multiple passaging [77]. Our previous work had clearly illustrated the need for passaging of cells in normoglycaemic conditions to prevent engendering a refractory state; unlike cloned cells, primary cells lose there endothelial cell characteristics through passaging, preventing habituation to normoglycaemia following early passaging in hyperglycamic medium, which is often the case for primary cells (e.g. HUVECs). In addition, cell culture experiments are always restricted by temporal constraints; the effects seen here were after only 50 h experimental procedures, whereas chronic exposures are likely to induce more substantial and potentially different impacts. Nevertheless, the fact that acute exposures caused such substantial effects in a model that reasonably mimics exposures that might be found in vivo supports the experimental design.

## Conclusions

This study highlights for the first time the effect of intermittent, physiologically relevant (as low as 0.4% of total carbohydrate load) fructose exposure on cultured vascular endothelial cells, and the interaction with concomitant glucose concentrations.

Exposure of EA.hy926 cells to a combination of fructose with glucose dramatically increased cellular respiration, while depressing NO production. The metabolic impacts in this regard are substantially greater than an equivalent increase in glucose alone, with the exception of proton leak, which was more clearly modulated by hyperglycaemic episodes and not seemingly impacted by fructose inclusion.

The findings could have profound implications with respect to dietary fructose intake, (e.g. free fructose, high-fructose corn syrup or sucrose). This is particularly important in the context of diabetes, where chronic hyperglycaemic is known to trigger endothelial dysfunction and increase atherogenic risk [50, 51]. The added complication of increased proton leak on top of modulation of metabolism and reduced NO generation induced by fructose could add to the long-term consequences of fructose ingestion in patients with diabetes. In addition, since fructose absorption is variable among different individuals [78, 79], monitoring the postprandial blood concentration of fructose as well as glucose may lead to more precise and meaningful, individual lifestyle interventions.

The next step in understanding these complex processes is to extend this study to primary human cells relevant to atherosclerotic risk, such as human coronary artery endothelial cells. These cells have been shown to express the transporter protein GLUT2 [80], which is responsible for fructose internalisation, and could be used to further explore the effects of fructose metabolisation on endothelial cell function and redox state. In addition, a proteomic approach could clarify the underlying mechanisms that drive the fundamental cellular changes observed.

## Abbreviations

ATP: Adenosine triphosphate
DMEM: Dulbecco’s Modified Eagle’s Medium
EGTA: egtazic acid
eNOS: endothelial nitric oxide synthase
FCCP: carbonyl cyanide-*4*-(trifluoromethoxy) phenylhydrazone
HG: high glucose
IG: intermediate glucose
MST: mitochondrial stress test
NO: nitric oxide
NMOCR: non-mitochondrial oxygen consumption rate
NG: normal glucose
OCR: oxygen consumption rate
ROS: reactive oxygen species
RNS: reactive nitrogen species
SOD: superoxide dismutase
